# Case method assisted implementation of guidelines on secondary prevention of coronary artery disease decreases mortality: a ten-year follow up of a randomized controlled study

**DOI:** 10.1186/1710-1492-6-S4-A6

**Published:** 2010-12-10

**Authors:** Anna Kiessling, Peter Henriksson

**Affiliations:** 1 Karolinska Institutet, Department of Clinical Sciences, Danderyd Hospital, Division of Cardiovascular Medicine, Stockholm, Sweden

## 

Previous findings show, two years after Primary Care Practitioners’ participation in an interactive pedagogic case-based learning program [1], that a significant reduction of blood lipid levels of their patients was achieved [2], and reported that the educational intervention was cost effective [3]. The aim of this study was to determine the size of any patient survival benefit from such interactive case-based learning method [1] aimed at facilitating implementation of guidelines in primary care.

This was a prospective randomized controlled trial in a primary care practice setting, in Stockholm, Sweden. New guidelines for secondary prevention in coronary artery disease were mailed to all general practitioners in the area and presented at a common lecture in 1995. The general practitioners were randomized according to their Primary Health Care Center into well-matched patient/physician pairs and were randomly allocated to active intervention with either exposure to a case-based learning method or usual care. General practitioners in the intervention group participated in recurrent case-based learning dialogues at their Primary Health Care Centers during a two-year period. A locally well-known cardiologist served as the facilitator. Consecutive patients (n=255) with coronary artery disease were included. Ten-year mortality rates were obtained from the *Cause of Death* register and were assessed as all cause and cardiovascular mortality.

The two Primary Health Care Center groups of patients and physicians were well matched and did not differ at baseline. The attendance rate at the seminars was 82% or higher. After ten years, nineteen (44%) of the patients included in the control group had deceased compared to 10 (22%) in the intervention group (p=0.017; log rank test). The inclusion of the covariates age, sex, hypertension, smoking and diabetes did not change the results. Patients treated by a specialist died at a rate comparable to the intervention group (23%). Cardiovascular mortality was 32% in the control group and 16% in the intervention group (p=0.007).

In conclusion, case-based learning methods for general practitioners improved survival in patients with coronary artery disease. The hazard ratio (HR) of survival between intervention and usual care is 0.45 (95% CI 0.20-0.95) if the case-based learning method is used to assist implementation of evidence based care.
